# Materia Medica Conference

**Published:** 1897-03

**Authors:** W. A. Dewey

**Affiliations:** Secretary; Ann Arbor, Mich.


### MATERIA MEDICA CONFERENCE.

The Committee on Materia Medica Conference presents the following programme for the meeting to be held in Buffalo, N. Y., Tuesday and Wednesday, June 22d and 23d, 1897:

GENERAL TOPIC.—Methods of purification of our Materia Medica.

First Session: Tuesday, June 22d, 1897. 3 P. M. "Does Critical Analysis and Drug Provings by the Chart Method mean too much Elimination." J. P. Sutherland, M. D., Boston, Mass., essayist.

*Disputants.*—A. L. Monroe, M. D., Louisville, Ky.; L. C. McElwee, M. D., St. Louis, Mo.; H. C. Allen, M. D., Chicago,

Ill.; A. C. Cowperthwaite, M. D., Chicago, Ill.; C. H. Evans, M. D., Chicago, Ill.; J. L. Moffatt, M. D., Brooklyn, N. Y.

Second Session: Tuesday, June 22d, 1897. 8 P. M. "Is the Method of the Baltimore Investigation Club Qualified to Fulfill its Purposes." Eldridge C. Price, M. D., Baltimore, Md., essayist.

*Disputants.*—George Royal, M. D., Des Moines, Ia.; Frank Kraft, M. D., Cleveland, Ohio; Pemberton Dudley, M. D., Philadelphia, Pa.; M. W. Van Denburg, M. D., New York; W. J. Hawkes, M. D., Chicago, Ill.; W. A. Dewey, M. D., Ann Arbor, Mich.

Third Session: Wednesday, June 23d, 1897. 10 A. M. "Purification by Means of Comparisons with Normal Standards." T. F. Allen, M. D., New York, essayist.

*Disputants.*—Conrad Wesselhœft, M. D., Boston, Mass.; M. Deschere, M. D., New York; J. C. Guernsey, M. D., Philadelphia, Pa.; E. H. Walcott, M. D., Rochester, N. Y.; J. B. G. Custis, M. D., Washington, D. C.; C. F. Meninger, M. D., Topeka, Kan.

The allottment of time fixed by the Institute at its last meeting for the appointed disputants is ten minutes each.

The remaining time in each session will be open to volunteer speakers, who shall be limited to purely extemporaneous remarks.

Each volunteer speaker will be allowed five minutes, as in the last Conference, and the utmost latitude as to time will be permitted *when the subject is adhered to*, but it will be strictly enforced against desultory and irrelevant remarks.

Those desiring to take part in this Conference, which promises to be of great interest, are urged to communicate at once with the Secretary, stating the topic upon which they desire to speak. This should be done at once. Last year many were shut out by sending in their names too late.

W. A. Dewey, M. D., *Secretary*.

Ann Arbor, Mich., January 15th, 1897.

